# Intercellular Communication between Hepatic Cells in Liver Diseases

**DOI:** 10.3390/ijms20092180

**Published:** 2019-05-02

**Authors:** Keisaku Sato, Lindsey Kennedy, Suthat Liangpunsakul, Praveen Kusumanchi, Zhihong Yang, Fanyin Meng, Shannon Glaser, Heather Francis, Gianfranco Alpini

**Affiliations:** 1 Richard L. Roudebush VA Medical Center, Indianapolis, IN 46202, USA; keisato@iu.edu (K.S.); linkenn@iu.edu (L.K.); sliangpu@iu.edu (S.L.); pkusuman@iu.edu (P.K.); yangjoe@iu.edu (Z.Y.); mengf@iu.edu (F.M.); heafranc@iu.edu (H.F.); 2 Indiana Center for Liver Research, Division of Gastroenterology & Hepatology, Department of Medicine, Indiana University School of Medicine, Indianapolis, IN 46202, USA; 3 Department of Medical Physiology, Texas A&M University, Temple, TX 76504, USA; SGlaser@medicine.tamhsc.edu

**Keywords:** liver fibrosis, extracellular vesicles, hepatocytes, macrophages

## Abstract

Liver diseases are perpetuated by the orchestration of hepatocytes and other hepatic non-parenchymal cells. These cells communicate and regulate with each other by secreting mediators such as peptides, hormones, and cytokines. Extracellular vesicles (EVs), small particles secreted from cells, contain proteins, DNAs, and RNAs as cargos. EVs have attracted recent research interests since they can communicate information from donor cells to recipient cells thereby regulating physiological events via delivering of specific cargo mediators. Previous studies have demonstrated that liver cells secrete elevated numbers of EVs during diseased conditions, and those EVs are internalized into other liver cells inducing disease-related reactions such as inflammation, angiogenesis, and fibrogenesis. Reactions in recipient cells are caused by proteins and RNAs carried in disease-derived EVs. This review summarizes cell-to-cell communication especially via EVs in the pathogenesis of liver diseases and their potential as a novel therapeutic target.

## 1. Introduction

### 1.1. Hepatic Cells

**Figure 1:**
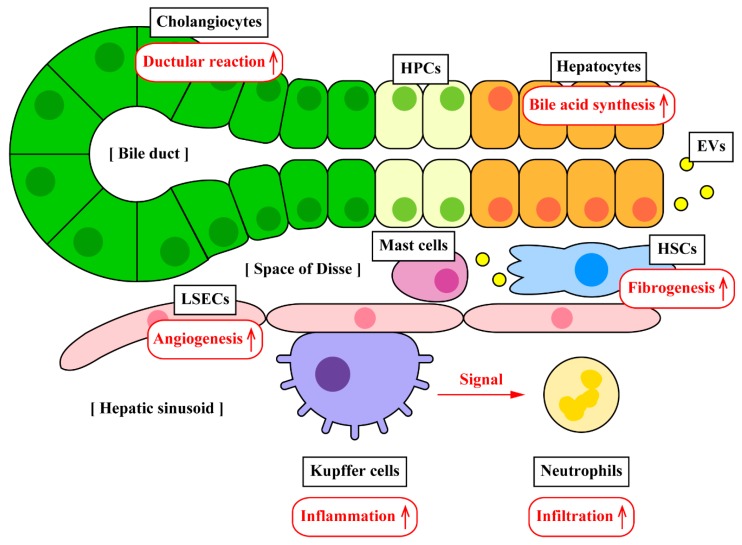
The location of liver cells and their orchestration in liver diseases. The liver consists of various cell types and they communicate and regulate with each other by secreting mediators. Previous studies have demonstrated that EVs carrying mediators are secreted from cells and transferred into other cells. EV-mediated cell-to-cell communication may play an important role in the pathogenesis of liver diseases. EVs: Extracellular vesicles; HPCs: Hepatic progenitor cells; HSCs: Hepatic stellate cells; LSECs: Liver sinusoidal endothelial cells.

The liver consists of various types of cells with the majority of hepatocytes (~70% of liver cell population) that form the parenchyma of the liver [1]. Other liver cells include intrahepatic cholangiocytes, Kupffer cells which are liver-resident macrophages, hepatic progenitor cells (HPCs) that are referred to as oval cells, liver sinusoidal endothelial cells (LSECs), and hepatic stellate cells (HSCs) (Figure 1). Liver diseases are initiated by the orchestration of hepatic cells. For example, cholangiopathies are bile duct disorders; however, not only cholangiocytes but also Kupffer cells are involved in the pathogenesis of cholestatic liver injury [2]. In non-alcoholic fatty liver disease (NAFLD) and its severe form non-alcoholic steatohepatitis (NASH), there is an interplay between hepatocytes, macrophages, and HSCs although detailed mechanisms of the orchestration of these cells are not well defined [3,4]. Upon activation during injury, cells secrete various mediators such as cytokines, chemokines, and hormones, which may act upon other neighboring cells in a paracrine fashion and trigger the disease progression. Previous review articles have summarized more information about the interplay between hepatic cells via cytokines and chemokines during liver injury [5,6].

### 1.2. Extracellular Vesicles

**Figure 2:**
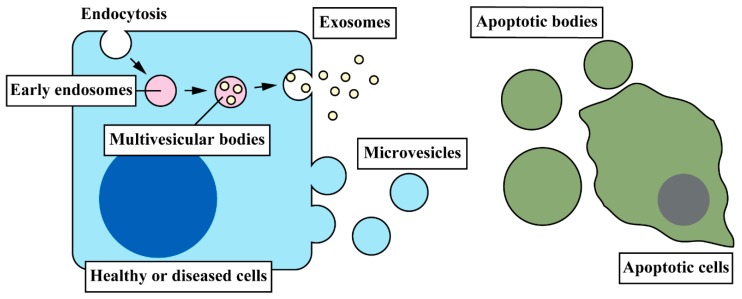
Three classes of EVs and their biogenesis. The smallest class of EVs, exosomes, is formed in multivesicular bodies, which are later endosomes carrying small vesicles. Multivesicular bodies are fused with the plasma membrane releasing exosomes. Microvesicles are formed directly from the membrane by outward budding. Exosomes and microvesicles carry cargo mediators, such as proteins, DNAs, and RNAs, which regulate cell events in recipient cells during EV-mediated cell-to-cell communication. Apoptotic bodies are the largest class of EVs and are released from apoptotic cells due to the destruction of cytoskeleton.

Extracellular vesicles (EVs), which are membrane-bound particles, play an important role in cell-to-cell communication during liver diseases [7,8]. EVs are currently classified into three classes: Exosomes, microvesicles or microparticles, and apoptotic bodies, according to their biogenesis. Exosomes are the smallest particles (~100 nm in diameter) formed within the endosomal network. Multivesicular bodies, which are endosomes containing internal vesicles, fuse with the plasma membrane and release those vesicles that are referred to as exosomes. Microvesicles (0.1–1 μm) are produced by the outward budding and fission of the plasma membrane. Apoptotic bodies (1–4 μm) are the largest vesicles in three classes that are released from apoptotic cells. In the process of apoptosis, cytoskeleton becomes destructive inducing outward budding from cell membranes. Figure 2 represents three classes of EVs. For more information of biogenesis and biological properties of EVs, see a previous systematic review [9].

Apoptotic bodies contain parts and debris of dying cells, and they are engulfed and recycled by phagocytic cells, such as macrophages. Exosomes and microvesicles contain various proteins, DNAs, and RNAs, and those vesicles are released from donor cells and can be transferred into recipient cells. Cargos carried in these vesicles are delivered into recipient cells resulting in the regulation of cell events by donor cells [10,11]. Since it is technically challenging to distinguish and isolate only exosomes or microvesicles, many previous studies utilize the mixture of these two types of EVs although it is possible that cargos differ between exosomes and microvesicles that are secreted from same cells. This review uses the term “EVs” including both exosomes and microvesicles, summarizing current understandings of cell-to-cell communication in liver diseases especially EV-mediated communication among liver cells.

## 2. Intercellular Communication in the Pathogenesis of Liver Diseases

### 2.1. Macrophages in Liver Inflammation

Kupffer cells are liver-resident macrophages that are essential for hepatic homeostasis [6]. When activated by danger signals, Kupffer cells recruit other immune cells such as monocytes and neutrophils to counteract diseased conditions initiating inflammation, fibrosis, angiogenesis, and repair [12,13]. Activated macrophages in the liver, either Kupffer cells or bone marrow-derived macrophages differentiated from infiltrated monocytes, secrete various mediators to activate other liver cells such as hepatocytes or HSCs leading to the pathogenesis of liver diseases. For example, macrophages secrete proinflammatory cytokines, interleukin (IL)-6 and IL-1β, during infection, and IL-6 can induce cholangiocyte proliferation leading to ductular reaction [14,15]. Macrophages also secrete profibrogenic transforming growth factor beta 1 (TGF-β1), a potent activator of HSCs leading to liver fibrosis [16]. These findings suggest that liver macrophages play a central role in the pathogenesis of liver diseases, thus leading to liver inflammation and fibrosis.

Macrophages also have a great phagocytotic ability and can internalize EVs [17]. Li et al. have demonstrated that EVs isolated from hepatocellular carcinoma (HCC) cell lines contain elevated levels of long non-coding RNAs (lncRNAs) TUC339 compared to EVs from normal hepatocyte line L-02 cells [18]. Monocytic line THP-1 cells internalized these HCC-derived EVs, and increased TUC339 levels in THP-1 cells were associated with macrophage M1/M2 polarization [18]. A recent study has demonstrated that induction of endoplasmic reticulum stress in HCC cell lines induces secretion of EVs that contain abundant miR-23a-3p, and HCC-derived EVs induce elevated expression of programmed death ligand 1 (PD-L1) in macrophages in vivo and in vitro leading to T-cell dysfunction and impaired proliferation [19]. These studies suggest that liver macrophages not only initiate signals but also internalize EVs as recipient cells to regulate their functions during HCC development and progression.

Macrophages also play a key role in alcohol-induced liver injury [20]. Alcohol abuse induces liver inflammation and damage followed by liver fibrosis [21]. HCC cell line HepG2 cells overexpressing alcohol metabolizing enzyme cytochrome P450 2E1, can secrete an elevated number of EVs following ethanol stimulation, which then can activate THP-1 cells through the CD40 ligand leading to their differentiation into inflammatory M1 phenotype [22]. Activated M1 macrophages in turn secrete several proinflammatory cytokines including IL-1β, IL-6, and tumor necrosis factor alpha (TNFα) and perpetuate the inflammatory process [23]. EVs can also be secreted from THP-1 cells and human primary monocytes upon ethanol exposure, and these THP-1 EVs can induce differentiation of naïve monocytes into the anti-inflammatory M2 phenotype by delivering cargo miR-27a [24]. EVs can be isolated from the serum of ethanol-fed mice. [25]. The alcohol-derived circulating EVs can induce the expression of M1 markers CD68 and TNFα but the suppressed M2 marker CD163 in murine macrophage line RAW 264.7 cells, suggesting that circulating EVs following ethanol feeding activate macrophages predominantly as the M1 phenotype leading to liver inflammation [25]. Neutrophils are activated by macrophages leading to infiltration during the inflammatory responses [26,27]. A previous study has demonstrated that patients with recent excess alcohol drinking have elevated numbers of circulating EVs that contain mitochondrial DNA (mtDNA), which are correlated with increased numbers of peripheral neutrophils [28]. The authors have utilized a mouse model of chronic plus binge alcohol drinking and have demonstrated that mice with alcohol abuse have elevated numbers of peripheral neutrophils and circulating mtDNA-enriched EVs compared to the control mice, and those EVs are hepatocyte-derived. Injection of EVs isolated from mice with chronic plus binge alcohol treatment into mice with chronic without binge alcohol drinking increased numbers of circulating lymphocytes, neutrophils, and monocytes [28]. Although it is unclear if injected EVs are internalized by liver macrophages and subsequently lead to lymphocyte recruitment or into neutrophils initiating infiltration, these findings suggest the important role of EVs in regulating neutrophil infiltration and inflammation in alcohol-induced liver injury.

Cell-to-cell interaction via EVs also plays a role in the pathogenesis of NASH. Hirsova et al. have demonstrated that lipotoxicity induced by incubation with lysophosphatidylcholine (LPC) induces elevated EV secretion from primary hepatocytes in vitro [29]. LPC-derived hepatocyte EVs as well as serum EVs isolated from mice fed with a high saturated fat, high fructose, and high cholesterol (HFFC) diet contained higher levels of IL-1β and IL-6 mRNAs compared to control EVs. These EVs can activate the expression of IL-1β and IL-6 in bone marrow-derived macrophages leading to liver injury [29]. In another study, stimulated hepatocyte-derived carcinoma cell line Huh7 cells and primary mouse hepatocytes with LPC secrete elevated numbers of EVs compared to those cells with vehicle, and LPC-derived EVs contain elevated levels of CXCL10 [30]. These lipotoxic EVs induced cell migration and activation of bone marrow-derived macrophages in a CXCL10-dependent manner [30]. Kakazu et al. have demonstrated that stimulation of immortalized mouse hepatocytes with palmitic acid increase EV secretion compared to vehicle, and these lipotoxic EVs are enriched with ceramide [31]. HFFC feeding elevated circulating EV numbers in mice and HFFC-derived EVs also contained elevated amounts of ceramide compared to EVs isolated from chow-fed mice [31]. Lipotoxic EVs induced migration of bone marrow-derived macrophages by delivering cargo ceramide [31]. These studies suggest that hepatocytes secrete EVs containing mediators during diseased conditions induced by alcohol or high fat diet, and hepatocyte-derived EVs regulate macrophage polarization leading to migration and cytokine production.

Drug-induced liver injury (DILI) is damaged liver conditions caused by exposure to toxic drugs. Exposure of hepatocytes to acetaminophen or galactatosamine increased EV secretion from hepatocytes in vitro, and administration of these drugs into mice caused liver damage and elevated numbers of circulating EVs in serum in vivo [32,33]. These drug-derived EVs contained elevated amounts of proteins and different protein profiles. Another study has demonstrated that exposure to acetaminophen increases levels of miR-122 carried in hepatocyte-derived EVs [34]. Since hepatocyte-derived EVs that contain elevated levels of miR-122 increase responses of THP-1 monocytes against lipopolysaccharide (LPS) [35], these studies suggest that hepatocytes release EVs that contain altered proteins and miRNAs to regulate the activation of monocytes and/or macrophages.

### 2.2. Hepatic Stellate Cells in Liver Fibrosis

HSCs are located in the space of Disse (Figure 1) and play an important role in hepatic fibrosis [36,37]. HSCs are normally in the quiescent state, however, they can transdifferentiate into myofibroblasts during the disease state [38,39]. Activated HSCs and myofibroblasts synthesize extracellular matrix (ECM) proteins including collagen type I, alpha smooth muscle actin (αSMA), and fibronectin leading to liver fibrosis. TGF-β1 is a profibrogenic polypeptide and is known to be associated with HSC activation and liver fibrosis. HSCs can be activated by internalization of EVs secreted from other cells or even from HSCs. Charrier et al. have demonstrated that HSCs secrete EVs that contain the connective tissue growth factor (CCN2) mRNA and protein, and those HSC-derived EVs are internalized into other HSCs delivering cargo CCN2 [40]. The active form of HSCs expresses elevated levels of CCN2 at diseased liver conditions, indicating the association between CCN2 and HSC activation [41]. Quiescent HSCs at normal conditions express high levels of Twist1 that inhibits CCN2 expression via miR-214, and EVs secreted from quiescent HSCs can suppress activation and fibrogenesis of other HSCs by delivering cargo Twist1 [41]. Platelet-derived growth factor (PDGF) is associated with migration and ECM production in myofibroblasts [42]. Kostallari et al. have demonstrated that EVs isolated from PDGF-BB-treated HSCs contain high levels of the PDGF receptor alpha (PDGFRα), and these PDGFRα-enriched EVs induce HSC migration in vitro and liver fibrosis in vivo [43]. These studies suggest that HSCs communicate with each other via EVs with different cargos regulating activation and fibrogenesis according to liver conditions.

Mast cells can be activated to release mediators by multiple triggers, and they play an important role in liver diseases [44]. Mast cell-deficient mice represent impaired HSC activation leading to attenuated liver damage and fibrosis during cholestatic liver injury, indicating the association between mast cells and pathogenesis of liver fibrosis via HSC activation [45]. Kim et al. have isolated serum EVs from patients with systemic mastocytosis and have found that these EVs contain high levels of mast cell signature proteins such as c-Kit [46]. These c-Kit-enriched EVs induced activation and expression of αSMA, collagen type I, and TGF-β1 by delivering cargo c-Kit in cultured HSCs in vitro, indicating EV communication between mast cells and HSCs at diseased conditions [46].

EVs from HepG2 cells or primary mouse hepatocytes treated with palmitic acid contain enriched miRNAs including miR-128-3p which can activate HSCs through the attenuated expression of peroxisome proliferator-activated receptor gamma (PPARγ) leading to liver fibrosis [47]. Another study isolated EVs from palmitic acid-treated Huh7 cells that can induce profibrogenic gene expression in cultured HSC line LX-2 cells indicating fibrogenic hepatocyte-to-HSC communication during NAFLD [48].

LSECs are located near HSCs (Figure 1), and crosstalk between LSECs and HSCs is associated with liver fibrosis [49]. Wang et al. have demonstrated that murine LSECs express elevated levels of sphingosine kinase 1 (SK1) during carbon tetrachloride (CCl_4_)-induced liver injury [50]. Cultured immortalized LSECs treated with triggering agents including PDGF and TGF-β1 secreted EVs carrying elevated levels of SK1 mRNA compared to the control. These SK1-enriched EVs induced AKT phosphorylation in human primary HSCs but not in HepG2 or THP-1 cells, indicating specific regulation against HSCs via LSEC-derived EVs [50]. Phosphorylation of AKT is associated with HSC activation and migration leading to liver fibrosis [51,52]. These findings support the crosstalk between LSECs and HSCs at diseased/fibrotic liver conditions.

Patients with the hepatitis C virus (HCV)-induced chronic hepatitis as indicated by alanine aminotransferase (ALT) > 100 IU/mL had higher numbers of EVs secreted from T cells in blood compared to healthy individuals or HCV patients with normal ALT levels (< 40 IU/mL) [53]. This study found that T cell-derived EVs regulated HSC activation and function, and effects of EVs varied depending on donor cells (CD4+ T cells or CD8+ T cells) or conditions of donor cells (apoptotic or not) [53]. Zhou et al. have isolated EVs from cultured HCC cell lines and demonstrated that HCC cell-derived EVs activate HSCs in vivo and in vitro leading to fibrogenesis by delivering cargo miR-21 which results in the activation of AKT in HSCs [54]. These studies suggest that various liver cells have crosstalk with HSCs coordinating for fibrogenesis during liver diseases.

### 2.3. Liver Sinusoidal Endothelial Cells in Angiogenesis

LSECs are endothelial cells located on the interface between blood cells and hepatocytes or HSCs (Figure 1). During liver fibrosis, expression levels of vascular endothelial growth factor (VEGF) are increased leading to proangiogenic action in LSECs and profibrogenic action in HSCs [55]. Angiogenesis in LSECs and fibrogenesis in HSCs and/or portal myofibroblasts are closely associated during disease progression. Isolated EVs from cultured portal myofibroblasts contain VEGF-A and can be internalized into LSECs inducing tube formation and proangiogenic responses [56]. A previous study has demonstrated using human umbilical vascular endothelial cells that HepG2 cells secrete EVs containing Vanin-1 during exposure to free fatty acids, and these HepG2-derived EVs drive tube formation and migration of endothelial cells by internalization [57]. The authors fed mice with a methionine- and choline-deficient (MCD) diet, which is a diet model of NASH, and found that MCD-fed mice had elevated numbers of circulating EVs containing Vanin-1 compared to control-fed mice. Vanin-1 enriched EVs induced tube formation and migration of endothelial cells, indicating crosstalk between hepatocytes and LSECs via EVs [57]. Liver fibrosis is also characteristic of cholestatic liver injury including bile duct ligation (BDL), which is a surgical ligation of the common bile duct that is widely used as an animal model of cholestasis and cholestatic liver injury [58]. Hedgehog signaling is essential for tube formation and angiogenesis of LSECs [59,60]. Witek et al. have isolated EVs from the serum and bile of BDL rats and have found that BDL-derived serum and biliary EVs contain elevated levels of Hedgehog ligands [61]. These BDL-derived EVs drove Hedgehog-dependent activation in LSECs, but EVs from healthy livers did not [61]. These findings suggest that LSECs and their proangiogenic actions are regulated by other liver cells via EVs.

### 2.4. Cholangiocytes in Ductular Reaction

Bile ducts consist of cholangiocytes (Figure 1) and cholangiocytes are associated with cholangiopathies, such as primary sclerosing cholangitis (PSC) [62]. Ductular reaction is the reactive biliary proliferation coupled with inflammation and is characteristic in several types of liver diseases [63]. Activated cholangiocytes secrete EVs during BDL leading to LSEC activation by delivering Hedgehog ligands as described previously [61]. Cellular senescence in cholangiocytes are also characteristic in PSC, and senescent cholangiocytes secrete senescence-associated secretory phenotype (SASP) markers such as IL-6, IL-8, and C-C motif chemokine ligand 2 (CCL2) leading to the activation of HSCs followed by fibrogenesis [64,65,66].

Cholangiocytes can be served as the recipient cells by internalizing EVs through the primary cilia [67,68]. EVs isolated from rat bile were incubated with cultured rat cholangiocytes, and they decreased cholangiocyte proliferation by the inhibition of ERK signaling in vitro, suggesting cholangiocyte regulation via EVs [67]. Deciliation of cholangiocytes decreased EV internalization and the associated regulation [67]. The abnormal accumulation of bacterial endotoxin or LPS in cholangiocytes has been found in liver tissues of patients with PSC [69]. Stimulation of human normal cholangiocyte line H69 cells with LPS increased EV secretion compared to vehicle, and these LPS-derived EVs induced enhanced cell proliferation as well as proinflammatory cytokine secretion including IL-1β, IL-6, and TNFα in other H69 cells in vitro [70]. These findings suggest that cholangiocytes communicate with each other via EVs at diseased conditions leading to cholangiocyte activation and proliferation.

### 2.5. Hepatocytes as Recipient Cells

As described previously, hepatocytes play a key role in various liver diseases as donor cells secreting EVs. During hepatocyte damage caused by alcohol or free fatty acid, hepatocytes secrete EVs leading to activation of macrophages and/or HSCs. Hepatocytes also internalize EVs as recipient cells and are regulated by other liver cells. Injection of EVs isolated from the alcohol-fed mice serum into naïve mice has demonstrated that injected EVs are internalized in primary hepatocytes causing elevated expression of CCL2 in vivo [25]. Li et al. have demonstrated that lncRNA H19 is highly expressed by cholangiocytes during cholestatic liver injury using *Mdr2^−/−^* mice, the mouse model for PSC [71]. The authors have found that cholangiocytes at diseased conditions secrete EVs containing H19, and cholangiocyte-derived EVs are internalized into hepatocytes suppressing small heterodimer partner by H19, which leads to increased bile acid synthesis resulting in cholestatic liver injury [72]. These studies suggest that hepatocytes communicate with other liver cells via EVs regulating their functions and vice versa.

## 3. Potential Utilization of Extracellular Vesicles

### 3.1. As Therapeutic Tools

Since EVs can regulate physiological events in recipient cells by delivering cargos, EVs may have potentials as a therapeutic tool for novel treatments of liver diseases. Transplantation of stem cells has demonstrated its therapeutic potential against liver diseases, especially liver fibrosis, using various sources of cells [73]. A clinical trial for transplantation of mesenchymal stem cells using patients with liver cirrhosis is currently ongoing (NCT03626090). Not only stem cells, but also stem cell-derived EVs may have therapeutic effects on liver diseases. Injection of EVs isolated from cultured human umbilical cord mesenchymal stem cells (hucMSCs) improved mouse liver conditions with CCl_4_-induced liver injury [74]. Previous studies have demonstrated that hucMSC-derived EVs have protective effects against oxidative stress, and these antioxidant effects are dependent on glutathione peroxidase1 carried in EVs [75,76]. Injection of human bone marrow mesenchymal stem cells (BM-MSCs) or EVs isolated from cultured BM-MSCs ameliorated CCl_4_-induced liver fibrosis by inhibiting Wnt/β-catenin signaling [77]. Injection of EVs isolated from mouse BM-MSCs improved liver conditions and survival rates in mice with galactosamine-induced DILI [78]. EVs isolated from human HPCs attenuated ductular reaction and liver fibrosis in PSC model *Mdr2^−/−^* mice by delivering cargo miRNA let-7 [79]. These studies suggest that stem cell-derived EV injection therapy can improve liver conditions and fibrosis during liver diseases. However, in most of the previous studies, EVs were isolated from cultured human stem cells and injected into model mice, which have a mismatch in species. In addition, it is unclear whether HPCs or other stem cells are activated during liver injury secreting therapeutic EVs in vivo. It is also undefined whether HPCs function as recipient cells to get activated by internalizing EVs secreted from other liver cells. Further studies are required to elucidate coordination and orchestration of liver cells in HPC-mediated liver repair.

Another approach for utilization of EVs as a therapeutic tool is to modify cargo mediators. Elevated expression of miR-155 in the liver has been reported in various liver diseases [80,81,82]. A previous study has demonstrated that electroporation loads miR-155 mimic into EVs isolated from murine B cells, and these miR-155 enriched EVs induce elevated CCL2 expression during LPS stimulation in Kupffer cells isolated from the miR-155 knockout mice [83]. Electroporation also loaded miR-155 inhibitor into B cell-derived EVs and those EVs were taken up by RAW 264.7 macrophage lines inhibiting TNFα secretion during LPS stimulation by delivering cargo miR-155 inhibitor [84]. Electroporation may be able to load not only mimics or inhibitors of miRNAs but also therapeutic chemicals and drugs, indicating the possible potentials of EVs as a drug carrier although current studies are limited and techniques are still not efficient [85]. Although further studies are required, these findings suggest that EVs can be a novel therapeutic tool as a mediator or drug carrier for the treatments of liver diseases.

### 3.2. As Diagnostic Tools

EVs contain proteins and RNAs, and those cargos can be cell- or disease-specific, indicating that the analysis of EV cargos may identify biomarkers leading to novel diagnostic techniques for liver diseases. Cholangiocarcinoma (CCA) is a bile duct cancer, and PSC patients often develop CCA in the later stage [86,87]. A previous study has characterized protein contents in EVs isolated from patients with PSC, CCA, or HCC, and healthy individuals [88]. EVs isolated from serum samples of CCA patients contained elevated levels of various proteins, such as CRP, PIGR, and AMPN, compared to those from other groups, and the receiver operating characteristic analyses represented that those candidate biomarkers could be useful for the diagnosis of CCA [88]. Another study has cultured patient-derived cells using collected HCC tissues from patients and characterized migration abilities for each cell to compare EV cargos between slow and fast migration groups [89]. This study identified various miRNAs carried in EVs that have a correlation and association with HCC cell migration, indicating that the analysis of EV miRNAs may be useful to predict cancer migration and progression [89]. These studies suggest that EVs secreted from cells at diseased conditions contain specific cargos, and the analysis of those cargo biomarkers could lead to the development of novel diagnosis or prediction of liver conditions. Numbers of previous studies have identified various candidate biomarkers carried in EVs. For more information of EV biomarkers in liver diseases, see previous reviews [7,8,90,91,92].

## 4. Conclusions and Perspectives

Different types of liver cells communicate with each other via EVs, and the orchestration of various cells plays an important role in the development and progression of liver diseases. The majority of studies are based on hepatocytes as donor cells with EV secretion and macrophages as recipient cells with EV internalization. This is probably because of the large population of hepatocytes (~70% total liver cells) and the phagocytotic ability of macrophages; however, other previous studies have identified various liver cells as donor and recipient cells. Future studies will reveal more detailed mechanisms of the orchestration of various liver cells mediated by EVs at the diseased conditions.

Liver functions can be regulated by EVs originated from other organs; this means that the physiological events in liver cells may be regulated by cells of other organs via secretion of EVs. Injected EVs via the tail vein are distributed into various organs. The majority of injected EVs are delivered into the liver but also into the spleen, intestine, and lung, and slightly into the pancreas and kidney [93]. This suggests that circulating EVs in serum could affect multiple organs, especially the liver. The liver and intestine coordinate in enterohepatic bile acid circulation and metabolism, and hepatic and intestinal cells influence each other by secretion and absorption of bile acids through bile acid receptors [94]. Gut microbiota influence liver conditions and may play an important role in liver diseases [95]. It is highly likely that functions of specific types of liver cells are regulated by mediators carried in EVs that are secreted from other organs or foreign organisms such as gut bacteria. Future studies will reveal more detailed mechanisms of EV-mediated interorgan communication in liver diseases.

In conclusion, different types of liver cells communicate with each other by secreting EVs and transferring cargo mediators into recipient cells leading to pathogenesis during diseased liver conditions. EVs and their cargos can be a therapeutic target to ameliorate cell functions in liver diseases.

## Abbreviations

ALT	alanine aminotransferase
αSMA	alpha smooth muscle actin
BDL	bile duct ligation
BM-MSCs	bone marrow mesenchymal stem cells
CCA	Cholangiocarcinoma
CCL2	C-C motif chemokine ligand 2
CCl_4_	carbon tetrachloride
CCN2	connective tissue growth factor
DILI	drug-induced liver injury
ECM	extracellular matrix
EVs	extracellular vesicles
HFFC	high saturated fat, high fructose, and high cholesterol
HCC	hepatocellular carcinoma
HCV	hepatitis C virus
HPC	hepatic progenitor cells
HSCs	hepatic stellate cells
hucMSCs	human umbilical cord mesenchymal stem cells
IL	Interleukin
lncRNAs	long non-coding RNAs
LPC	Lysophosphatidylcholine
LPS	Lipopolysaccharide
LSECs	liver sinusoidal endothelial cells
MCD	methionine- and choline-deficient
mtDNA	mitochondrial DNA
NAFLD	non-alcoholic fatty liver disease
NASH	non-alcoholic steatohepatitis
PDGF	platelet-derived growth factor
PDGFRα	PDGF receptor alpha
PD-L1	programmed death ligand 1
PPARγ	peroxisome proliferator-activated receptor gamma
SASP	senescence-associated secretory phenotype
PSC	primary sclerosing cholangitis
SK1	sphingosine kinase 1
TGF-β1	transforming growth factor beta 1
TNFα	tumor necrosis factor alpha
VEGF	vascular endothelial growth factor
